# Hypertension after renal transplantation

**DOI:** 10.1007/s00467-007-0627-7

**Published:** 2009-05-01

**Authors:** Tomáš Seeman

**Affiliations:** Department of Pediatrics and Transplantation Center University, University Hospital Motol, Charles University Prague, Second School of Medicine, V Úvalu 84, 15006 Prague, Czech Republic

**Keywords:** Hypertension, Children, Renal transplantation, Graft function, Graft survival, Blood pressure control, Angiotensin-converting enzyme inhibitors

## Abstract

Hypertension is a common and serious complication after renal transplantation. It is an important risk factor for graft loss and morbidity and mortality of transplanted children. The etiology of posttransplant hypertension is multifactorial: native kidneys, immunosuppressive therapy, renal-graft artery stenosis, and chronic allograft nephropathy are the most common causes. Blood pressure (BP) in transplanted children should be measured not only by casual BP (CBP) measurement but also regularly by ambulatory BP monitoring (ABPM). The prevalence of posttransplant hypertension ranges between 60% and 90% depending on the method of BP measurement and definition. Left ventricular hypertrophy is a frequent type of end-organ damage in hypertensive children after transplantation (50–80%). All classes of antihypertensive drugs can be used in the treatment of posttransplant hypertension. Hypertension control in transplanted children is poor; only 20–50% of treated children reach normal BP. The reason for this poor control seems to be inadequate antihypertensive therapy, which can be improved by increasing the number of antihypertensive drugs. Improved hypertension control leads to improved long-term graft and patient survival in adults. In children, there is a great potential for antihypertensive treatment that could also result in improved graft and patient survival.

## Introduction

Hypertension is a common and serious complication in adult as well as in pediatric patients after renal transplantation [1–3]. It is an important risk factor for cardiovascular morbidity and mortality in transplanted patients [4, 5]. Furthermore, it is a strong risk factor for impaired graft survival in adult and pediatric patients [6–10]. In recent years, there has been an increasing interest in posttransplant hypertension management, as it is a potential tool for improving long-term graft and patient survival.

## How should blood pressure be measured in transplant patients?

Blood pressure (BP) must be measured during every outpatient visit as casual BP (CBP) according to the recommendations of the National High Blood Pressure Education Program (NHBPEP) Working Group on High Blood Pressure in Children and Adolescents [11]. However, CBP has its limitations, mainly in that it can neither distinguish between true and white-coat hypertension (i.e. increased CBP in the presence of physician or nurse but normal ambulatory BP) nor measure BP during nighttime and sleep. It has been shown in several studies that ambulatory BP monitoring (ABPM) is a better method for evaluating BP than is CBP measurement in children after renal transplantation [12–14]. The main reasons are the ability of ABPM to reveal white-coat hypertension and to measure BP during nighttime. Furthermore, ABPM is superior to CBP in regard to better correlation with target organ damage, such as left ventricular hypertrophy (LVH) [15] and the ability to diagnose masked hypertension, i.e. normal CBP but increased ambulatory BP [12]. Finally, the results of ABPM are more closely related to renal function in transplanted patients than are the results of CBP [16]. Therefore, regular use of ABPM is recommended in all patients after renal transplantation regardless of CBP. How frequently ABPM should be used in transplanted children is not clear. However, it is evident from the superiority of ABPM over CBP that ABPM should be performed at least once a year in every transplanted child. Most devices used in pediatrics are based on oscillometry. However, no ABPM device has been validated by the British Hypertension Society for use in children. Nevertheless, they are widely used in children, and the most commonly used is the oscillometric SpaceLabs device [12–15].

Home BP self-measurement is also an important method of BP measurement. It is increasingly used as a valuable supplement to CBP and ABPM in children with chronic renal failure or on renal replacement therapy [17, 18]. It can improve hypertension control and patient antihypertensive therapy compliance [19]. Therefore, it is especially recommended in children on antihypertensive medication.

## Prevalence of posttransplant hypertension

The prevalence of hypertension in children after renal transplantation ranges considerably, between 58–89% [2, 3, 12, 20, 21–28]. The results of several published studies are summarized in Table 1. The reason for the wide range in the prevalence of hypertension is based mainly on the different methods of BP measurement and different definitions of hypertension in various trials. Studies that used CBP measurements always reported lower prevalence of hypertension than do studies that used ABPM. This phenomenon clearly emphasizes the importance of ABPM, as it also measures BP during the night when BP is often increased in transplanted patients. An interesting finding of several studies [23, 25, 28] is the predominance of nighttime hypertension in these patients. If a child after renal transplantation has hypertension, it is usually at nighttime, either isolated nighttime or combined with daytime hypertension. This finding further stresses the importance of ABPM with its to monitor BP values during the night, which are usually elevated in hypertensive children. The predominance of nighttime over daytime hypertension in transplanted children was confirmed most recently in a study by McGlothan et al. [29].

**Table 1:** Prevalence of hypertension in children after renal transplantation

Author	Method of BP measurement	Definition of HT	Number of patients	Prevalence of HT
Baluarte et al. 1994 [2]	Casual BP	Use of antihypertensive drugs regardless of BP level	277	59%
Sorof et al. 1999 [3]	Casual BP	Use of antihypertensive drugs regardless of BP level	5251	58%
Lingens et al. 1997 [21]	ABPM	BP >95th centile for clinic BP or use of drugs	27	70%
Giordano et al. 2000 [23]	ABPM	BP >95th centile	37	62%
Sorof et al. 2000 [24]	ABPM	BP load >25% (95th centile for clinic BP)	42	83%
Morgan et al. 2001 [25]	ABPM	BP >95th centile for clinic BP and BP load >30%	45	62%
Serdaroglu et al. 2005 [26]	ABPM	BP >95th centile for ABPM and BP load >30%	26	73%
Seeman et al. 2006 [28]	ABPM	BP >95th centile for ABPM or use of drugs	36	89%

*BP* blood pressure, *ABPM* ambulatory blood pressure measurement, *HT* hypertension

Some studies using ABPM defined hypertension using the 95th percentile for CBP [24, 25] and other studies only on the basis of antihypertensive drugs use without taking the current BP level into account [2, 3]. Furthermore, some studies defined hypertension on the basis of current BP level without taking antihypertensive drugs use into account and therefore did not separate patients with normal BP without antihypertensive drugs (i.e. patients with spontaneous normotension) from patients already treated, i.e. patients with controlled hypertension [22]. Moreover, different studies used different normative data for evaluating ABPM [12, 23, 28]. If strict criteria are used for defining hypertension—i.e. all children on antihypertensive drugs and all children with elevated current BP are defined as hypertensive—then the prevalence of ambulatory hypertension reaches 90% [28]. The use of these strict criteria is important for clinical practice. Children should be defined as hypertensive on the basis of both criteria: use of antihypertensive drugs and current BP level. The main reason is the fact that it has been shown in several trials that transplanted patients with controlled hypertension have the same graft survival as spontaneous normotensive patients (i.e. normal BP without antihypertensive drugs). In contrast, patients with uncontrolled hypertension have significantly worse graft survival [10, 30–32]. Therefore, using antihypertensive drugs as the only criterion for defining hypertension without knowing the current BP level would lead to misinterpretation of the importance of the influence of BP on the overall prognosis of transplanted patients.

## Nondipping phenomenon

Reduced physiological decrease of BP during the night (nocturnal dip) is a frequent finding in children after renal transplantation. The definition of the nondipping phenomenon in pediatrics is still not clearly given. Usually, the adult definition is used (i.e. nighttime decrease of systolic and/or diastolic BP < 10%) or decrease of mean BP < 5.5% [21]. In the general pediatric population, the nondipping phenomenon is a highly specific marker of secondary hypertension [33]. In transplanted children, it has been revealed in 30–72% of patients [21, 24, 25, 28]. The reason for the frequent nondipping phenomenon in transplanted patients is not fully understood. Several factors, such as treatment with steroids and calcineurin inhibitors, sodium retention, impaired renal function, chronic rejection, or renal artery stenosis, have been discussed [21, 24, 25, 34]. Adult transplant patients who are nondippers have greater left ventricular mass than do dippers [35]. However, in a pediatric study, no significant difference in the left ventricular mass index between children with normal and attenuated nocturnal BP dip was found [28].

## Causes of hypertension

**Fig. 1:**
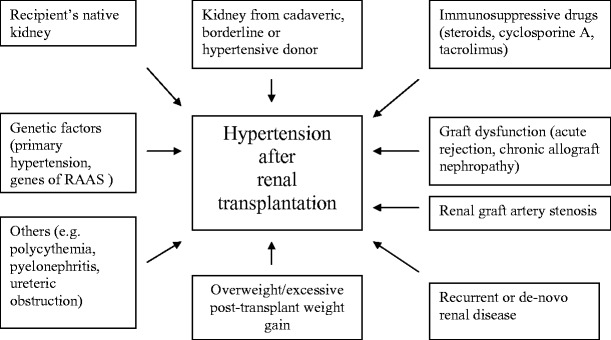
Causes of hypertension in patients after renal transplantation. *RAAS* renin-angiotensin-aldosterone system

The etiology of posttransplant hypertension is clearly multifactorial [2, 3, 20, 36]. Causes and risk factors for development of hypertension after renal transplantation are summarized in Fig. 1.

## Hypertension prior to transplantation, primary renal disease, and native kidney nephrectomy

Hypertension prior to transplantation caused mainly by the diseased native kidney is believed to be a risk factor for the presence of hypertension after successful renal transplantation [28, 31, 36–39]. Furthermore, several small studies reported that native kidney nephrectomy prior to renal transplantation protects against the persistence of hypertension after renal transplantation or that BP decreases after native kidney nephrectomy in transplanted patients [23, 37, 40, 41]. However, this issue is a point of contention, as there are no large trials confirming the findings from these small, single-center studies.

## Donor source

Children receiving kidneys from deceased donors are more frequently hypertensive than are children receiving grafts from living donors [3, 36]. The beneficial effect of organs from living donors on recipient BP could be related to fewer immunological injuries at the vascular and parenchymal sites, to shorter cold ischaemia time of the graft, smaller ischaemia–reperfusion injury, or other factors associated with living-donor transplantation. It can be speculated that the lower prevalence of hypertension among children after living-donor transplantation could be one reason for better graft survival of the living-donor grafts in comparison with cadaveric donor grafts. This hypothesis is supported by the results of a single-center study that shows that posttransplant hypertension is, together with episodes of acute rejection, the only independent determinant of graft survival in children after living-donor transplantation [42].

## Immunosuppressive medication

### Steroids

The etiological role of steroids in posttransplant hypertension is well known [34, 43]. Several factors, such as sodium retention or increase in cardiac output and renal vascular resistance, induce steroid-related hypertension. Elimination of steroids in stable patients showed BP reduction in adult and pediatric patients [43–45], and children with steroid-avoidance immunosuppressive protocol showed improvement in hypertension [46]. Sarwal et al. demonstrated that children with steroid-free primary immunosuppression show significantly improved hypertension [46]. In a cross-sectional study, patients on alternate-dose steroid treatment showed significantly lower prevalence of hypertension than did children on daily steroid medication [28]. This is in good agreement with other studies showing that conversion from daily to alternate-dose steroid therapy significantly reduces BP in adults as well as in children [47, 48]. Therefore, steroid-sparing or steroid-free immunosuppression are possible treatment strategies for improving BP control in transplanted children.

### Calcineurin inhibitors

With the introduction of the calcineurin inhibitor cyclosporine, there has been a dramatic increase in the prevalence of posttransplant hypertension [36, 49]. Hypertension induced by cyclosporine is caused by several mechanisms. Preglomerular afferent arteriole vasoconstriction, sodium and water retention, increased sympathetic nerve activity, renin system stimulation, and endothelial cell dysfunction with impaired endothelium-derived relaxing-factor (nitric oxide)-dependent vasodilatation are the most important mechanisms [50, 51]. Gordjani et al. [36] showed in their large, single-center study on 102 children that high trough levels of cyclosporine (>400 ng/ml) were associated with a significantly higher incidence of hypertension in comparison with children with levels < 400 ng/ml (91% vs 57%).

The newer calcineurin inhibitor tacrolimus also has hypertensinogenic effects similar to cyclosporine. In the only randomized controlled trial comparing cyclosporine and tacrolimus-based immunosuppression in pediatric renal transplanted patients, there were no significant differences in the prevalence of hypertension between children treated with cyclosporine and those with tacrolimus [52]. The same was also true in a pediatric cross-sectional study [28] and in the US FK506 Kidney Transplant Study in adults [53]. However, some other studies in adults showed less hypertension in patients on tacrolimus than in those on cyclosporine [54, 55], and therefore, this issue remains controversial.

### Newer immunosuppressive drugs

Newer immunosuppressive agents such as mycophenolate mofetil, sirolimus, or everolimus do not have BP-increasing effects, and therefore, their use is a further option to improve hypertension control in transplanted children [51].

### Graft dysfunction (acute rejection, chronic allograft nephropathy)

There is a dual relationship between BP and graft dysfunction. On the one hand, graft dysfunction elevates BP, whereas on the other hand, elevated BP accelerates graft function decline. This is reflected by the aphorism “the kidney: the culprit and the victim”. In adults, impaired graft function is associated with elevated BP and increased risk of hypertension [7, 16, 31, 32, 56–58]. In a single-center study, Mitsnefes et al. found no difference in mean calculated glomerular filtration rate (GFR) or acute rejection episodes between normotensive and hypertensive children [10]. However, hypertensive children had poor allograft function (GFR < 50 ml/min per 1.73 m^2^) more frequently than did normotensive patients, whereas children with normal BP more frequently had normal graft function (GFR > 75 ml/min per 1.73 m^2^). On the contrary, in another pediatric study using CBP measurements, Gordjani et al. could find no differences in graft function between normotensive and hypertensive patients [36]. In our single-center, cross-sectional study we found no significant differences between normotensive and hypertensive children in graft function or presence of biopsy-proven chronic rejection. Nevertheless, due to the dual relationship between BP and allograft function, it is difficult to demonstrate whether posttransplant hypertension is more the cause or the result of graft dysfunction.

### Weight gain, overweight, and obesity

Current body weight or change in body weight is a well-known and potent determinant of BP level in adults and children [59, 60]. Most children gain weight after renal transplantation [61]. The main reason is the use of high-dose steroids in the early posttransplant period and long-term maintenance treatment with these “obesitogenic” drugs. Studies in adult and pediatric patients have shown that increased body mass index (BMI) or obesity in transplanted patients is associated with decreased long-term graft function and graft survival [61–63]. Furthermore, in the report of the North American Pediatric Renal Trials Cooperative Studies (NAPRTCS), obese children aged between 6 and 12 years have higher risk for death than do nonobese patients of the same age [61]. Therefore, body-weight control should be recommended in all children after renal transplantation to improve BP control and decrease the risk of cardiovascular events.

### Stenosis of transplant renal artery

Stenosis of the graft artery has become a rare cause of hypertension with the current surgical technique of using aortic patches. In the past, retrospective studies from pediatric transplantation centers diagnosed renal artery stenosis in up to 9% of children. Currently, however, with the use of a donor aortic patch, the rate of stenosis at the anastomosis decreased down to 0% [64, 65]. Doppler ultrasonography and magnetic resonance angiography are noninvasive techniques that can easily diagnose this cause of hypertension. The treatment of choice is percutaneous transluminal angioplasty; surgery should be reserved for cases of angioplastic failure [66].

## Recurrent or de novo renal disease

The development of recurrent or de novo glomerulonephritis [e.g. focal segmental glomerulosclerosis (FSGS), IgA nephropathy, membranoproliferative glomerulonephritis, lupus nephritis, atypical hemolytic uremic syndrome) may be associated with the occurrence of hypertension, although these conditions are not common causes of significant posttransplant hypertension. The development of glomerulonephritis in the graft, mainly that caused by FSGS, usually presents as massive proteinuria, with hypertension being a less common feature. Treatment focuses primarily on the primary disease (e.g. plasmapheresis in case of the recurrence of FSGS). However, antihypertensive therapy, especially with angiotensin-converting enzyme (ACE) inhibitors, are believed to influence positively the course of recurrent or de novo renal disease in the graft [67, 68].

## Clinical consequences of hypertension in transplant recipients

### Consequences to renal allograft

**Fig. 2:**
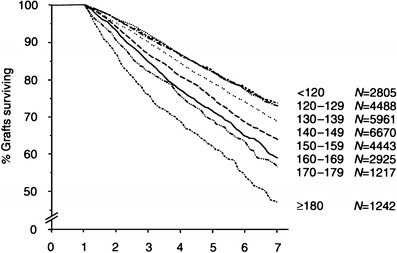
Association of renal allograft survival with recipient systolic blood pressure (courtesy Prof. Opelz, Heidelberg, for the Collaborative Transplant Study) on X axis years after renal transplantation, adult and pediatric renal transplant recipients together

**Fig. 3:**
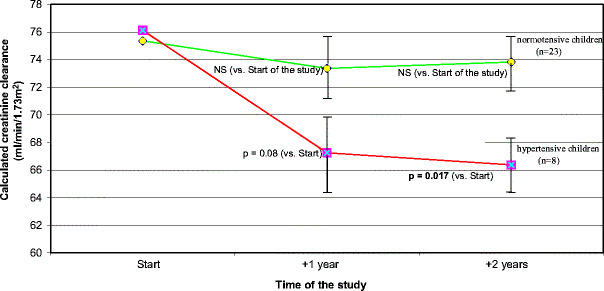
Graft function in children who where normotensive or hypertensive at 2 years (values of SD in *error bars* are divided by 10)

Hypertension is a strong predictor for graft loss. The most robust evidence comes from the results of the large, multicenter Collaborative Transplant Study (CTS) published by Opelz et al. [6], which showed a linear negative relationship between CBP and renal graft survival (Fig. 2). This is true not only for adults but also for children between 0 and 18 years of age (unpublished results from CTS, Prof. Opelz (2007), Heidelberg, personal communication). This relationship between BP and graft survival was later confirmed by many other studies in adult and pediatric patients [5, 7, 10, 69, 70]. Results from the NAPRTCS registry showed that the use of antihypertensive medication, a definition for hypertension in this retrospective analysis, is associated with higher graft failure [3]. Other smaller, and usually single-center, studies showed that patients with higher BP had lower graft survival than did patients with lower BP. Increased BP is therefore clearly associated with decreased graft survival. This association is linear and is evident even in the normotensive range. Despite these clear findings, it is still a matter of debate whether posttransplant hypertension is a real cause of chronic allograft dysfunction or only the result of renal dysfunction or both. Several findings from retrospective studies such as that of Mitsnefes et al. [10] that show that hypertension is associated with allograft failure in children with normal graft function but not in children with severely impaired graft function suggest that hypertension is not only a marker of graft dysfunction but also a direct cause of renal graft damage. This deleterious effect of high BP is well established in chronic nephropathies of native kidneys. Furthermore, in a more recent study, it was shown that children who remained hypertensive during a 2-year interventional trial on BP control lost significant graft function compared with children in whom BP was lowered to the normotensive range [71] (Fig. 3).

## Cardiovascular consequences of hypertension

Similar to the general population, hypertension is associated with increased cardiovascular morbidity in the transplanted patient population. LVH is a frequent type of cardiac end-organ damage in hypertensive children after renal transplantation, occurring in 50–82% of them [22, 25, 28, 72]. Matteucci et al. [22] found a correlation between LVM index (LVMI) and mean 24-h systolic BP. However, a more recent study done by Morgan et al. [25] could not find any relationship between LVMI and ambulatory BP data. Similarly, Mitsnefes et al. and our group found no correlation between LVMI and BP [28, 72], although Mitsnefes et al. measured only CBP. The results of these studies support a hypothesis that actual BP level is not the principal risk factor of LVH in transplanted patients and that many other factors, such as the type of immunosuppressive and antihypertensive therapy, pretransplant cardiac anomalies, anemia, obesity or physical activity also play a role in the etiology of cardiac abnormalities after transplantation [22, 72]. However, a recent study Kitzmueller et al. found a correlation between LVMI and ABPM data at repeated measurement but not at baseline, suggesting that BP control, i.e. change in BP level during longitudinal follow-up, is important to maintain the myocardial architecture [73].

Hypertension is also a risk factor for increased cardiovascular mortality seen in transplanted adult patients [4]. Similar studies in children are rare. The Dutch Cohort Study demonstrated that hypertension also is one of the most powerful risk factor for cardiovascular morbidity and mortality in pediatric patients after renal transplantation [74]. In this study cardiovascular events were the most common cause of death and hypertensive children had a 3-times higher risk of overall mortality than normotensive children.

## Treatment of posttransplant hypertension

There is clear evidence from observational studies on the correlation between BP and cardiovascular morbidity, mortality, and graft function that posttransplant hypertension must be treated at least as it is in the general pediatric population. Most other issues related to treating hypertension in children after renal hypertension are less clear.

### Which drug(s) should we use?

There are no studies comparing different classes of antihypertensive drugs in children after renal transplantation. Therefore, it is not known whether one class of drugs is better than another in transplanted patients. Historically, calcium-channel blockers (CCB) have been considered the drugs of choice for posttransplant hypertension because they counteract the afferent arteriolar vasoconstriction caused by calcineurin inhibitors and reduce their nephrotoxicity [1, 75, 76]. Furthermore, in a double-blind randomized trial on adult patients, it was shown that after 2 years, long-acting nifedipine improved allograft function more effectively than did the ACE inhibitor lisinopril [77]. However, one can still speculate as to whether or not the increased GFR induced by CCB will have detrimental long-term effects on allograft survival by increasing glomerular filtration due to predominant afferent arteriolar vasodilation. Longer randomized controlled trials are needed to answer this question.

There has been some concern that ACE inhibitors or angiotensin-receptor blockers (ARB) may deteriorate graft function in a case of graft-artery stenosis or due to the preferential efferent arteriolar vasodilation and reduction of intraglomerular pressure. However, it has been recently demonstrated that ACE inhibitors are safe and efficient in adult and pediatric transplanted patients [78, 79]. Furthermore, ACE inhibitors and ARB can slow progression of chronic native kidney diseases in adults mainly by long-term reduction of intraglomerular pressure [80]. Data on the renoprotective ability of ACE inhibitors in children are still lacking. The ability of ACE inhibitors to slow progression of chronic allograft nephropathy (CAN), which is the most common cause of late graft loss [81], has never been proven in a prospective interventional trial on adult or pediatric patients. Some retrospective studies have shown promising results, such as stabilization or even an improvement in patient survival and graft function in patients with CAN [79, 82, 83]. However, results from CTS published recently showed no improvement in patient or graft survival with ACE-inhibitor treatment [84]. Therefore, this issue is still controversial and needs prospective interventional trials to be resolved.

Beta-blockers are also effective drugs in transplanted patients. Hausberg et al. showed that atenolol is an effective and safe antihypertensive drug in transplanted patients [85]. However, beta-blockers are not able to reduce proteinuria as ACE inhibitors do. A further disadvantage of beta-blockers is their negative metabolic effects (increased lipid levels or impaired glucose tolerance), which may further contribute to the increased risk of cardiovascular disease in these patients.

Sodium retention is often present after renal transplantation, and therefore, diuretics are important antihypertensive drugs in these patients as well. Thiazide diuretics should be preferred in patients with normal graft function, whereas loop diuretics should be given in patients with impaired graft function. Diuretics may also have detrimental metabolic effects, such as hyperlipidemia, hyperuricemia, or hyperglycemia. Potassium-sparing diuretics are used rarely due their risk of hyperkalemia.

There are no data on the use of ARB in children after renal transplantation. However, in adults, there are several small, single-center studies showing that ARB can also be used in transplanted patients [86].

All four major classes of antihypertensive drugs can therefore be used in transplanted patients. Posttransplant hypertension has a multifactorial etiology and is often severe; therefore, a combination therapy of two to three antihypertensive drugs is usually needed to control it. Which drug should be used as a first-line treatment remains the individual decision of the physician because it has not been consistently shown that one class is better than another in renal transplant recipients [1, 76]. In most pediatric renal transplantation centers, the most commonly used antihypertensive drugs are CCB, which are given to 38–65% of transplanted children [14, 24–26, 28, 29]. The second most commonly prescribed drugs are ACE inhibitors and beta-blockers. Diuretics are given less frequently. Data on the prescription of antihypertensive drugs in different pediatric studies are summarized in Table 2.

**Table 2:** Classes of antihypertensive drugs used in children after renal transplantation

Study	Percentage of all treated patients in the study	Calcium-channel blockers (CCB)	Beta blockers (BB)	ACE inhibitors/angiotensin-receptor blockers	Diuretics	Others (alpha-blockers, clonidine)
Sorof et al. 2000 [24]	76%	62%	33%	5%	21%	0%
Morgan et al. 2001 [25]	49%	49%	n.d.	0%	n.d.	0%
Serdaroglu et al. 2005 [26]	62%	62%	0%	0%	0%	0%
Seeman et al. 2006 [28]	86%	58%	64%	19%	39%	0%
McGlothan et al. 2006 [29]	59%	38%	23%	24%	0%	3%
Ferraris et al. 2007 [14]	75%	65%	23%	12%	0%	12%

*n.d.* not determined

Nonpharmacological lifestyle measures (reduction of increased body weight, reduction of salt intake, physical activity) should be encouraged, even during antihypertensive drug therapy, as they target the risk factors not only for hypertension but also for cardiovascular morbidity and mortality (obesity, increased salt intake, physical inactivity).

### What is the target BP in transplanted children?

What the target BP should be for patients after renal transplantation is still a matter of debate. The National Kidney Foundation Task Force on Cardiovascular Disease recommends a target BP level < 130/85 for adult renal allograft recipients and < 125/75 for proteinuric patients, similar to guidelines for hypertension management in patients with diabetic nephropathy [87]. However, there are no prospective interventional trials showing that target BP lower than the conventional cutoff of 140/90 will improve graft function and long-term graft survival. The same is true for pediatric renal transplant recipients. Furthermore, there are no data demonstrating that, even in chronic native kidney diseases, more aggressive hypertension treatment can slow chronic renal insufficiency progression in children. The results of an ongoing European multicenter study [Effect of Strict Blood Pressure Control and ACE Inhibition on the Progression of Chronic Renal Failure in Pediatric Patients (ESCAPE) trial] will answer this question. The current recommendation of the fourth report of the NHBPEP Working Group on High Blood Pressure in Children and Adolescents recommends target BP < 90th percentile for children with chronic kidney diseases [11]. Although no such recommendation has yet been made for managing hypertension after renal transplantation, adoption of this target would seem logical [88].

### How is hypertension controlled in transplanted children?

Only a minority of children treated for hypertension after kidney transplantation have BP at least below the target level recommended for the healthy population, i.e. < 95th centile [23, 26, 28]. Therefore, antihypertensive therapy in children after renal transplantation is insufficient, leading to poor BP control. The prevalence of persistent hypertension despite antihypertensive treatment (i.e. prevalence of uncontrolled hypertension) ranged between 45% and 82% in recent pediatric studies using ABPM [21, 23, 25, 26, 28]. This means that only 18–55% of children after renal transplantation had hypertension controlled by drugs with BP at least < 95th centile. Data on hypertension control from several cross-sectional studies are summarized in Table 3. The reasons for the insufficient antihypertensive therapy in transplanted patients have not been thoroughly investigated. Many factors, such as chronic allograft dysfunction, need for lifelong use of BP-elevating immunosuppressive drugs (steroids, cyclosporine, tacrolimus), obesity, salt retention, renin secretion from diseased native kidneys, and the fear of ACE inhibitors in transplanted patients are discussed as the major reasons for inadequate BP control in transplanted patients [1, 51]. However, there is increasing evidence from several recent studies that posttransplant hypertension is not truly therapy resistant but is not appropriately treated. In studies with a low number of prescribed antihypertensive drugs, there was a higher prevalence of uncontrolled hypertension [14, 24–26, 28, 29] (Table 3). The results of a recent retrospective study on ACE inhibitors in children further emphasizes this concept [79]. The authors could achieve adequate hypertension control in 100% of transplanted children 1 year after addition of an ACE inhibitor in patients with refractory hypertension using CBP measurement. Whether the use of ACE inhibitors can improve BP control in a prospective study still needs to be investigated. Current results on posttransplant hypertension control highlight a high potential for improved antihypertensive therapy in children after renal transplantation. The key issue is whether the poor hypertension control can be improved. A recent prospective interventional trial on intensified treatment of hypertension showed that ambulatory BP could be significantly reduced after 2 years by increasing the number of antihypertensive drugs, especially ACE inhibitors and diuretics [71].

**Table 3:** Hypertension (HT) control in children after renal transplantation using ambulatory blood pressure monitoring (ABPM)

Author	Definition of uncontrolled HT	Number of treated patients (% of all patients)	Prevalence of uncontrolled HT	Mean number of antihypertensive drugs per treated patient
Lingens et al. 1997 [21]	Only daytime BP >95th centile for ambulatory BP despite use of drugs	17 (63%)	65% (11/17)	n.d.
Giordano et al. 2000 [23]	Daytime, nighttime, or 24-h BP >95th centile despite use of drugs	25 (68%)	72% (18/25)	1.5
Morgan et al. 2001 [25]	Daytime BP >95th centile for clinic BP or nighttime BP >95th centile for clinic BP minus 10% despite use of drugs	22 (49%)	82% (18/22)	1.4
Serdaroglu et al. 2005 [26]	Daytime or nighttime BP >95th centile for ABPM and BP load >30% despite use of drugs	16 (62%)	81% (13/16)	1.0
Seeman et al. 2006 [28]	Daytime or nighttime BP >95th centile despite use of drugs	31 (86%)	45% (14/31)	2.1

*n.d.* not determined

Lastly, noncompliance can play an important role, particularly in adolescents. Therefore, adherence to the recommended antihypertensive drugs should be checked during every outpatient visit. Furthermore, home BP measurement should be encouraged, as it increases therapeutic compliance in hypertensive patients [19].

### Can improved BP control improve graft survival?

There is enough evidence from studies on adults and children to show that hypertension is associated with decreased graft function and subsequent shorter graft survival [3, 5–7, 10, 73, 74]. However, it is still unclear whether posttransplant hypertension is a cause or only a marker of allograft dysfunction. Indirect evidence from recent retrospective studies demonstrates that increased BP is not only a marker but also a true cause of graft damage. Mitsnefes et al. have shown that hypertension was also associated with graft failure in children with well-preserved renal function, strongly suggesting, a causal relation between increased BP and poor graft survival [10]. Furthermore, Mange et al. demonstrated that the effect of elevated BP on graft function was independent of baseline allograft function in predicting poor long-term allograft survival [7]. Other studies have clearly shown that not “hypertension per se” (especially if defined only on the basis of the use of antihypertensive drugs) but the actual BP level (regardless of using antihypertensive drugs or not) is the decisive factor influencing graft function and survival [30–32, 56]. Several observational studies have demonstrated that patients with controlled hypertension (i.e. normal actual BP in a patient on antihypertensive medication) have similar graft survival to patients with spontaneous normotension that is significantly better than in patients with uncontrolled hypertension [30–32, 56]. However, there are no interventional studies on pediatric or adult patients showing that improved BP control in transplanted patients can improve graft survival. On the other hand, a recent retrospective study from the CTS demonstrated that improved BP control in the last seven years was independently associated with improved long-term graft survival [89]. Further smaller retrospective studies provided clear evidence that not hypertension “per se” (defined often on the basis of the use antihypertensive drugs regardless of actual BP level) but the actual BP level is the decisive factor influencing graft function and graft survival regardless of whether normal BP level is achieved by antihypertensive drugs or is spontaneous [31, 56]. These results provide a clinical rationale for rigorously controlling hypertension in transplanted patients. In a recent prospective interventional study on intensified hypertension control it was shown that hypertensive children in whom BP was lowered during a 2-year period of time to normotension had stable graft function, in contrast to children who remained hypertensive after 2 years and who lost significantly GFR [71] (Fig. 3). This is the first prospective interventional study showing that the hypertension control in transplanted children can be improved and that improved BP control can stabilize graft function.

### Can improved BP control improve patient survival?

It is well established that hypertension is a strong and independent risk factor for increased cardiovascular mortality seen in transplanted adult and pediatric patients [4, 74, 90]. And whereas no prospective interventional studies have been conducted to test the hypothesis that hypertension treatment can decrease cardiovascular mortality in this specific patient population, the recent retrospective study from the CTS showed that improved BP control was associated with improved long-term patient survival [89]. Patients whose systolic BP decreased from > 140 mmHg at 1 year to < 140 mmHg at 3 years posttransplant had a significantly lower risk of cardiovascular death than did patients whose systolic BP remained > 140 mmHg. These data, although derived retrospectively, are the most convincing to date and suggest that BP control, even if instituted late after renal transplantation, improves cardiovascular and graft outcome. The observed improvements in long-term graft survival and patient survival associated with improved hypertension treatment underline the potential value of aggressive hypertension control in all transplanted patients, even late after transplantation.

## Conclusions

**Fig. 4:**
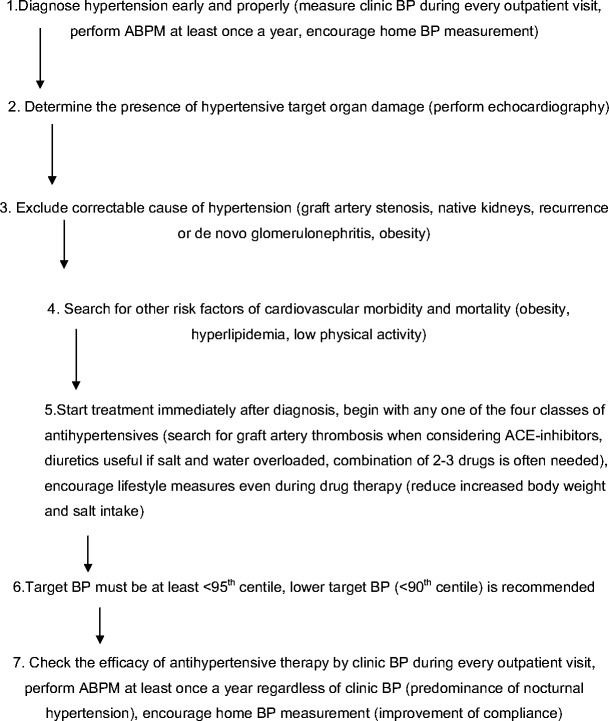
Algorithm on hypertension management after renal transplantation

Hypertension is a frequent complication in children after renal transplantation, with a prevalence ranging from 60% to 90%. It is an important independent risk factor for graft and patient survival. The etiology is multifactorial: pretransplant hypertension; damaged native kidneys; immunosuppressives such as steroids, cyclosporine, or tacrolimus; renal-graft artery stenosis; and chronic allograft nephropathy being the most common causes. By necessity, only CBP can be obtained at most routine outpatient visits. ABPM is the best method for BP evaluation in transplanted children and should be regularly performed in every transplanted child approximately once a year. Posttransplant hypertension can be treated with all classes of antihypertensive drugs, as no specific class has been shown to be better than the others in transplanted patients. The most commonly prescribed drugs are CCB. The rate of therapeutic hypertension control is unsatisfactorily low: only 20–50% of treated children have normal BP. This poor hypertension control can be improved by a more rigorous diagnostic and treatment approach, with regular use of ABPM and an increased number of prescribed antihypertensive drugs, especially of ACE inhibitors. A proposed algorithm for the management of hypertension in transplanted children is given in Fig. 4. There is compelling evidence that improved hypertension control is associated with improved graft and patient survival in the adult transplanted population. Similar data on children are based on several small, observational trials only and one prospective interventional trial that showed that improved hypertension control can stabilize graft function.

## Appendix

### Supported by the Ministry of Education of the Czech Republic VZ MŠMT No 0021620819 and Ministry of Health VZ MZ No 0006420301 and NR/9524-3.

**Questions**

Answers (only one correct for each question) appear following reference list.

1. How frequent is hypertension in children after renal transplantation?
  - 10–20%
  - 20–30%
  - 30–50%
  - 50–60%
  - 60–90%
2. What is the best method for blood pressure evaluation in transplanted children?
  - Casual blood pressure
  - Ambulatory blood pressure monitoring
  - Home blood pressure
  - All methods are equal
3. The main cause of posttransplant hypertension is:
  - Diseased native kidneys
  - Steroids and calcineurin inhibitors
  - Impaired graft function
  - All causes listed above
  - Volume overload
4. The consequences of hypertension after transplantation are:
  - Left ventricular hypertrophy and increased mortality
  - Improved graft function
  - Obesity
  - All of the above
  - Improved physical activity
5. The drugs of choice in posttransplant hypertension are:
  - ACE inhibitors
  - Beta-blockers
  - Calcium-channel blockers
  - Diuretics
  - No class of drugs is better than the other
6. How many transplanted children treated for hypertension reach normal blood pressure under therapy?
  - Nearly all
  - About 75%
  - 50–75%
  - Only 20–50%
  - Less than 10%
7. What are the measures of how to improve hypertension control?
  - Dietary measures
  - Reducing the dose of immunosuppressive drugs, especially mycophenolate mofetil
  - Increasing the dose of antihypertensive drugs above the recommended dosages
  - Increasing the number of antihypertensive drugs, especially ACE inhibitors and diuretics
  - Removing native kidneys in all transplanted children

**Answers:**

1. e
2. b
3. d
4. a
5. e
6. d
7. d
